# Supplementary material for: Seasonal Dynamics and Damage of Halyomorpha halys in Italian Vineyards
Source: Insects. 2024 May 22;15(6):378. doi: 10.3390/insects15060378 (PMC11203982; doi:10.3390/insects15060378)
Supplement: Supplementary file 1 [file insects-15-00378-s001.zip › insects-2974531-supplementary.pdf]

Supplementary file for:

Seasonal Dynamics and Damage of *Halyomorpha halys* in Italian Vineyards

Davide Scaccini *, Diego Fornasiero, Paola Tirello, Simone Vincenzi, Massimo Cecchetto, Ilirjan Allgjata, Carlo Duso and Alberto Pozzebon *

Department of Agronomy, Food, Natural Resources, Animals and Environment, University of Padova, Viale dell'Università 16, Legnaro, 35020 Padova, Italy

* Correspondence: davide.scaccini@unipd.it (D.S.); alberto.pozzebon@unipd.it (A.P.)

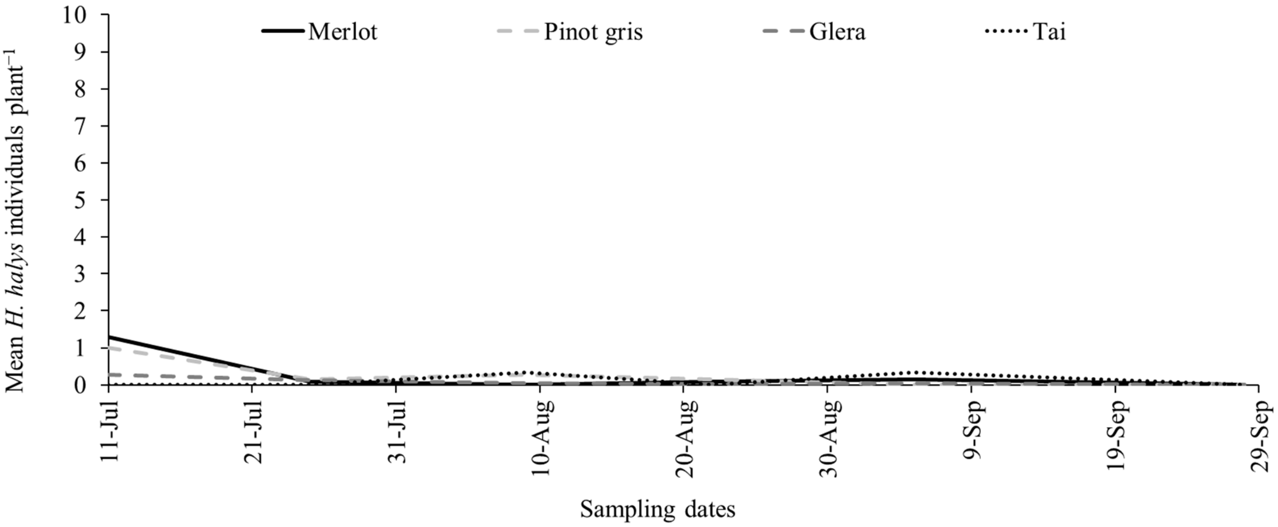

Figure S1. *Halyomorpha halys* seasonal dynamics on different grape cultivars observed from visual sampling in 2017.

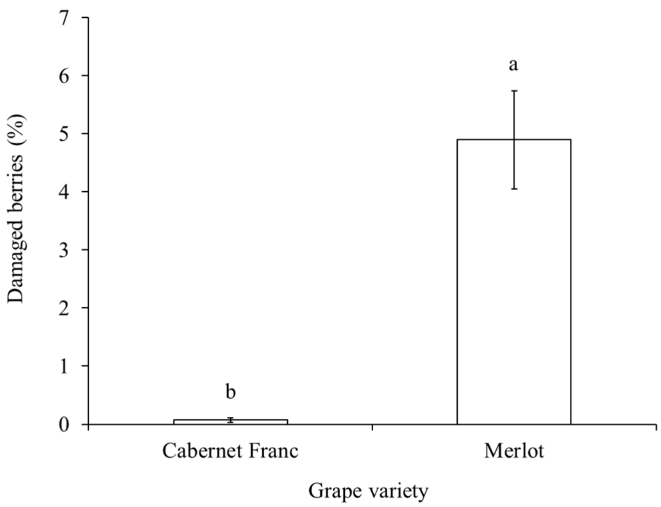

Figure S2. Percentage (mean ± std. err.) of berries with signs of *Botrytis cinerea* and sour rot in the red grapes Cabernet Franc and Merlot. Different letters indicate significant differences according to Tukey–Kramer's test (α = 0.05).

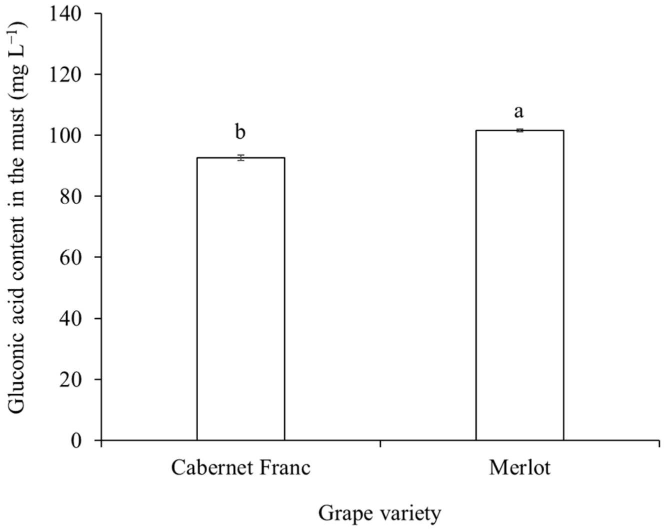

Figure S3. Glucose content (mean ± std. err.) in must of red grapes. Different letters indicate significant differences according to Tukey–Kramer's test (α = 0.05).
